# Expert System for Monitoring the Malaxing State of the Olive Paste Based on Computer Vision †

**DOI:** 10.3390/s18072227

**Published:** 2018-07-11

**Authors:** Diego M. Martínez Gila, Pablo Cano Marchal, Juan Gómez Ortega, Javier Gámez García

**Affiliations:** Robotics, Automation and Computer Vision Group, Electronic and Automation Engineering Department, University of Jaen, ES-23071 Jaen, Spain; pcano@ujaen.es (P.C.M.); juango@ujaen.es (J.G.O.); jggarcia@ujaen.es (J.G.G.)

**Keywords:** olive oil production process, computer vision, expert system, thermomixer variables

## Abstract

The malaxing of olive paste is one of the most important sub-processes in the virgin olive oil production process. The master continuously supervises the olive paste inside the themomixer to assess the preparation state of the olive paste and he acts manually over the process variables. The viscosity, granularity, and the presence of olive oil over the paste are the main indicators of the olive paste state. Furthermore, the temperature, time, coadjuvant addition and the shovel speeds are the process variables in the thermomixer. In this work, different image-processing parameters have been proposed to automatically assess the aforementioned indicators and they have been used as inputs in the designed fuzzy controller. Also, the outputs of this controller have been evaluated according to a sequence of images obtained inside the thermomixer and during the malaxing process in a real olive mill.

## 1. Introduction

**Figure 1:**
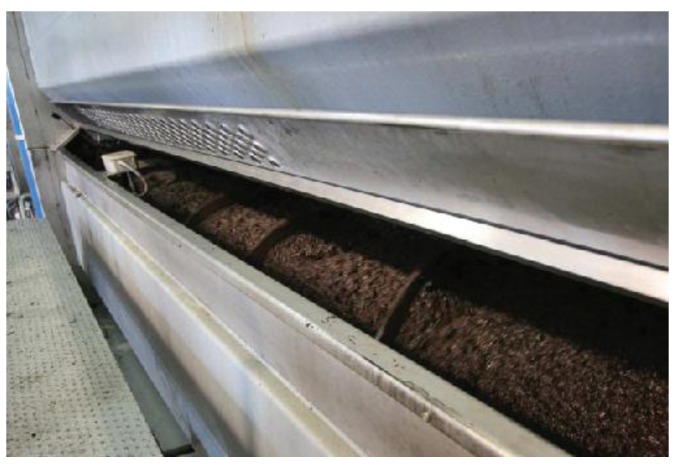
Industrial thermomixer in the virgin olive production process [7].

The production of olive oil has increased from 1.5 million of tons to 3 million of tons from 1990 to 2015. 90% of this quantity comes from Spain, Italy, and Greece according to the International Olive Council. There are more than 12,000 olive mills around the globe where virgin olive oil is produced and the production process is roughly the same everywhere: the farmer drops the olive fruits into the hoppers; the olives are conveyed to the crushing machine where they are turned into olive paste; the olive paste is kneaded within thermomixers (see Figure 1), that is, the malaxing process; subsequently the olive paste is pumped out to the horizontal centrifugal machine where olive oil is separated from olive skins and pits according to their densities; finally, the oil is filtered and stored in large cellars.

The malaxing process is one of the main phases within the production process and different technological variables can affect the quality and the quantity of the produced virgin olive oil. In this context, different works have studied the cause and effect relations between these variables and the olive oil features. In [1] the authors collected more than 80 articles and the first conclusion was that mixing conditions such as time, temperature and the composition of the atmosphere in the thermomixer can affect the activity of the enzymes that are responsible for the healthy and organoleptic properties of the product.

Over the years, different methodologies have been carried out to monitor and control biological processes based on intelligent systems and human knowledge [2,3]. In particular, in [4] the authors reviewed the application of fuzzy expert system approaches in food and beverage processes for quality evaluation, sensing and control.

In the context of the virgin olive oil production process, the use of advanced techniques to monitor and control processes is not so democratized, and only local systems exist to measure basic variables such as temperatures and flow rates [5]. Focusing on the malaxing process, the person responsible for the production plant acts as sensor. He periodically supervises the preparation of the olive paste inside the thermomixer and modifies the process variables (typically temperatures, rotation speed of the shovels, coadjuvant addition and time) according to his assessments.

The work documented in this paper has two aims. The first contribution is to propose a method to automatically assess the olive paste state inside the thermomixer based on computer vision and image processing. On the other hand, the second approach is to suggest the values of the process variables according to the olive paste elaboration state. Three variables were selected to assess this state [6]:

- FO: Floating oil. This variable is related to the presence of oil over the surface of the paste.
- V: Viscosity of the paste. High viscosities are related to difficult and non-prepared pastes.
- G: Granularity of the paste. Also, it denotes the olive paste state.

## 2. Materials and Methods

**Figure 2:**
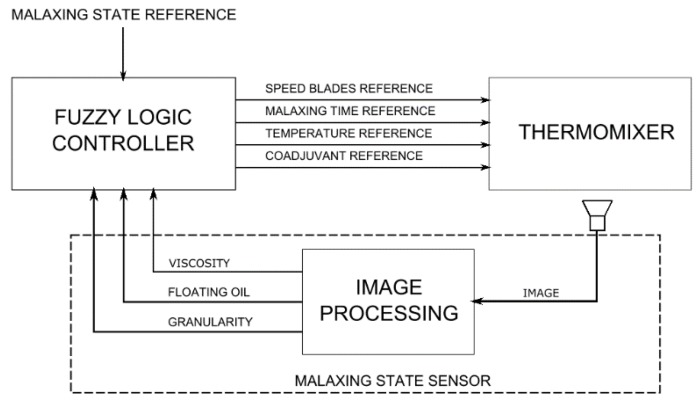
Flow chart of our approach.

The solution that has been proposed in this work is summarized in Figure 2. The first block is the malaxing state sensor. It has the acquired images at the input and three state variables as output (floating oil, granularity, and viscosity). According to the malaxing state reference, the second block—fuzzy logic controller—decides the rotation speed of the blades, the temperatures of the thermomixer, the quantity of coadjuvant to mix with the paste and the residence time.

### 2.1. Experimental Setup

The experiments were carried out with the hardware deployed in a real oil mill. Two experimental setups were installed. The first computer vision system was installed in the thermomixer to acquire online images. Furthermore, the second setup was located next to the thermomixer and it was employed to capture images at-line by sampling.

**Figure 3:**
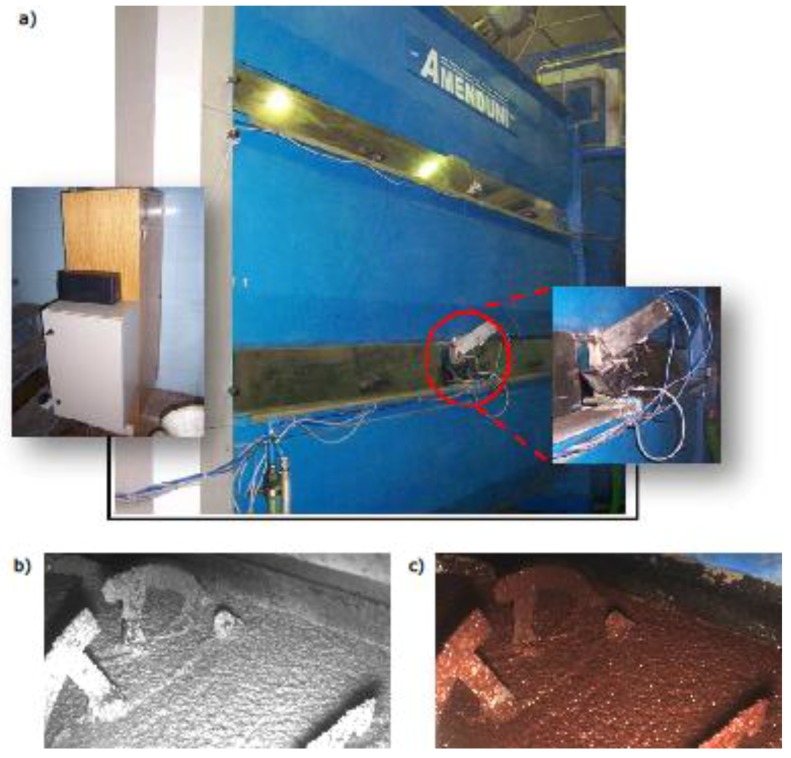
Subfigure (**a**) shows the vision camera and the lighting lamp monitoring the olive paste. At the left of this subfigure is the cabinet that houses the computer PC and the electronic associated; Subfigure (**b**) presents an example of image acquired by the infrared sensor; Subfigure (**c**) displays the same scene in the visible color channel.

Figure 3 shows an industrial three-body thermomixer located in the virgin olive oil production plant. The computer vision system was installed in the lower body to be operated conveniently. An industrial camera was enclosed in a metallic chamber to protect it from environmental dust. It was composed of a dual sensor (infrared and visible) vision system (JAI manufacturer, model AD-080 GE, Copenhagen, Denmark) equipped with a lens with 6 mm of focal length. The selected lighting system was a halogen lamp and near to this setup a computer with the acquisition and processing software was installed.

**Figure 4:**
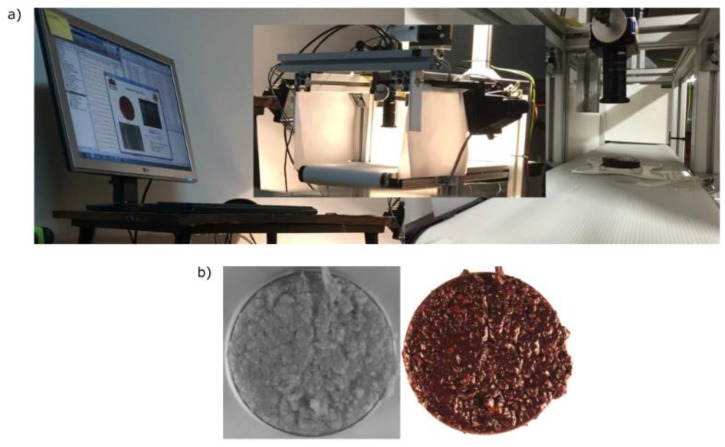
Image setup for at-line acquisitions. Subfigure (**a**) is composed by the vision camera, two halogen lamps and the conveyor belt. At the left of this subfigure appears the PC which handles the experimental setup. To the left of this subfigure is the Petri dish with a sample of paste below the vision device; Subfigure (**b**) displays the acquired images in the infrared (**left**) and color (**right**) channels.

Figure 4 presents the second experimental setup installed next to the thermomixer to acquire images at-line. It was composed of one JAI AD-080 GE camera and two 100 W halogen lamps. Also, a conveyor belt was employed to locate the sample within the field of view of the camera.

The first experimental setup was used to obtain the viscosity and floating oil determination, whereas the second one was employed to obtain the granularity parameter.

### 2.2. Viscosity Determination

The quantity of paste adhering to the blades inside the thermomixer relates to the viscosity of the paste (based on previous interviews with the operator of the production plant). Because of this issue the first image processing task was to extract this information.

**Figure 5:**
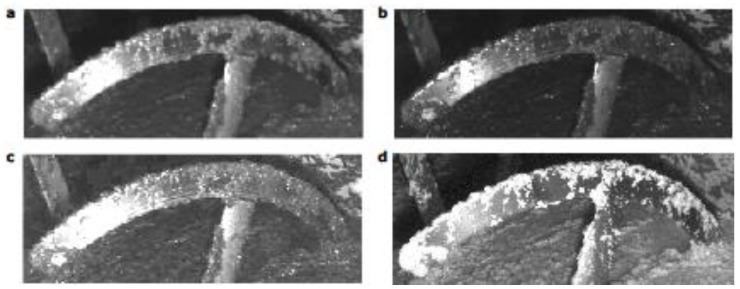
These figures show pates over the blades and inside the thermomixer, in the red channel (**a**); green channel (**b**); blue channel (**c**) and infrared channel (**d**). In the last one the olive paste is more contrasted.

A sequence of images was monitored from dirty to clean shovels. Figure 5 displays the first image of the sequence for each camera vision channel. It shows the difference of tones between the olive paste and the stainless steel blade. The difference is higher in the infrared channel. Then a binary mask was applied to consider only the shovel area as the region of interest. After that, the quantity of paste attached to the shovel was computed as the average intensity according to Equation (1). This operation was performed for each image of the sequence.

(1) $$\bar{I_{c}}=\frac{\sum_{n=1}^{N}\sum_{m=1}^{M}I_{C}\left(n,m\right)\;\times \;I_{mask}\left(n,m\right)}{N\cdot M}$$

where $I_{C}\left(n,m\right)$ denotes the gray value for each pixel in the C image channel (red, blue, green, and infrared); N and M are the number of rows and columns respectively; $I_{mask}\left(n,m\right)$ is the designed mask where the pixels out of the shovel have a value of zero and the pixels which match with the shovel have a value of 1.

### 2.3. Floating Oil Determination

The infrared information was useful to extract the quantity of olive oil floating over the semi-solid paste because different materials or products with the same visible color have different absorption indexes in the infrared spectral band. Then the region of interest was selected to obtain a sequence of olive paste surface images most likely to have floating oil and without the presence of shovels.

**Figure 6:**
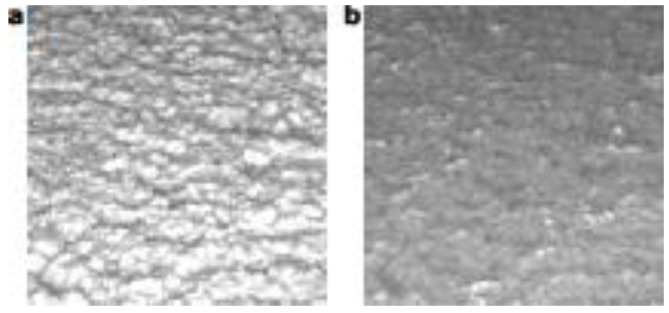
Subfigures (**a**,**b**) are cropped image with presence and absence respectively of floating olive oil over the olive paste [7].

As in the previous parameter, a sequence of images was acquired from absence to presence of floating oil. Figure 6 shows two images, the first and the last images of the sequence. Also, in this case Equation (1) was applied to obtain the average intensity for each color channel.

### 2.4. Olive Paste Granularity Determination

The texture of the olive paste inside the thermomixer can be influenced by the movement of the shovels and by the presence of floating oil too. It could be fixed with a structural change in the thermomixer (e.g., by removing one shovel). Therefore, an experimental setup was built at-line to evaluate the granularity of the olive paste surface.

A batch with 4000 kg of olives was milled and then malaxed for 120 min. The purpose of this experiment was to obtain an evolution of olive pastes from unprepared to prepare in excess. Three samples of olive paste were manually taken from the thermomixer every 10 min. A total of 36 samples were collected and prepared in Petri dishes.

The setup was composed of the dual sensor vision device and images in the infrared (IR) and visible color space. The Petri dish shape was the region of interest for each image. For this region of interest (ROI), the spatial distribution of pixels in the whole image region was useful to carried out statistical analysis and firstly the gray level co-occurrence matrix (GLCM) was computed [8,9,10]. Furthermore, it was normalized by applying Equation (2).

(2) $$P_{c,n,m}\left(i,j\right)=\frac{p_{c}\left(i,j\right)}{I\cdot J}$$

where I·J is the sum of all matrix elements; c denotes the color channel (red, green, blue, or infrared); n is the number of sample during the malaxing process (from 1 to 12); m is the number of the repeated sample (from 1 to 3).

Based on the GLCM matrix, different statistical parameters were extracted such as contrast, correlation and homogeneity were obtained from this co-occurrence matrix. Then, the granularity of the olive paste was studied according to Equations (3)–(5).

(3) $$C_{c,n,m}=\underset{i,j}{\sum }{\left|i-j\right|}^{2}P_{c,n,m}\left(i,j\right)$$

(4) $$\;E_{c,n,m}=\underset{i,j}{\sum }P_{c,n,m}{\left(i,j\right)}^{2}$$

(5) $$\;H_{c,n,m}=\underset{i,j}{\sum }\frac{P_{c,n,m}\left(i,j\right)}{1+\left|i-j\right|}$$

where $C_{c,n,m}$ is the contrast parameter and it returns a measure of the intensity contrast between a pixel and its neighbour over the whole image and it is zero for a constant image; $E_{c,n,m}$ is the energy parameter and it returns the sum of squared elements in the GLCM; $H_{c,n,m}$ is the homogeneity parameter and it returns a value that measures the closeness of the distribution of elements in the GLCM matrix to the matrix diagonal. It is 1 for a diagonal GLCM. The final goal of this experiment was to evaluate which parameter is the most sensitive to tiny changes in lump sizes over the olive paste surface.

### 2.5. Fuzzy Controller Design

**Figure 7:**
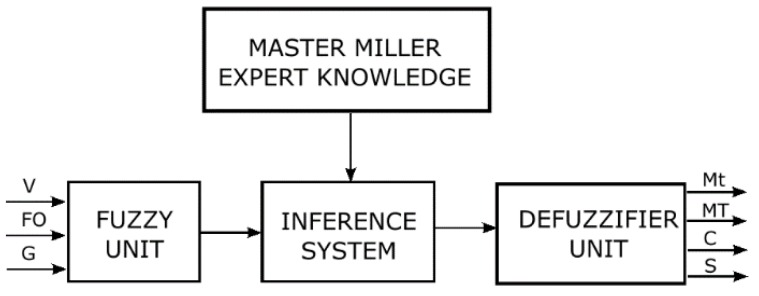
Fuzzy Rule-Based System. Viscosity, floating oil, and granularity (V, FO, and G respectively) are the inputs. Malaxing time, malaxing temperature, coadjuvant addition and speed of the shovels (Mt, MT, C and S respectively) represents the outputs.

The second approach of this work was to design the control system to suggest the value of the process variables. The flow chart of this control system appears in Figure 7. It was built with four blocks, that is, master miller expert knowledge, fuzzy unit, inference system and defuzzifier unit. The master miller knowledge was based on previous interviews with different plant operators and their knowledge was modelled to build the inference system rules. The inference system was configured following Mamdani’s method [11] and the input and output membership functions were adjusted manually with the Fuzzy Logic Toolbox of MATLAB.

**Figure 8:**
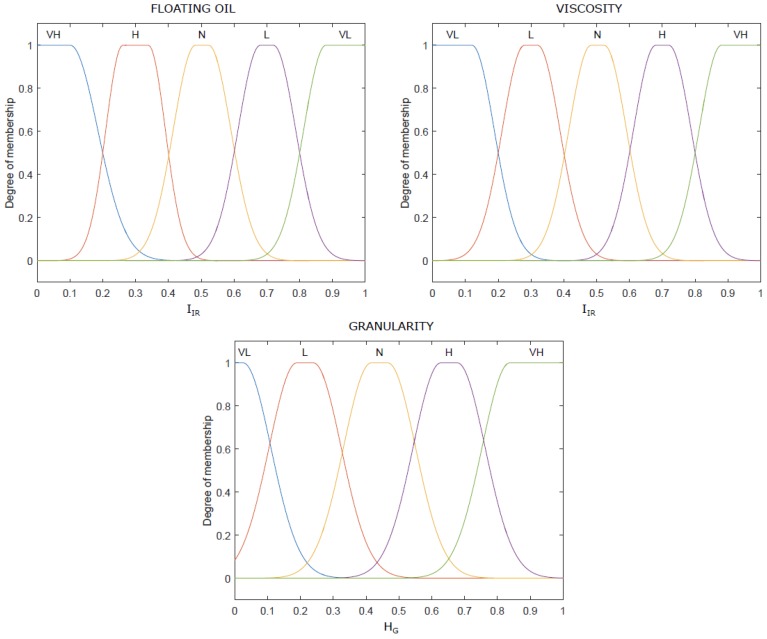
Input fuzzy membership functions.

The membership functions of the fuzzy unit are presented in Figure 8. Three variables were selected as inputs: olive paste granularity obtained according to the GLCM matrix, presence of floating oil based on IR vision and the quantity of paste over the shovels.

Thus, the universe of discourse for each variable obtained from the image processing step was scaled from 0 to 1 relating to the minimum and maximum value for each parameter. In addition, to relate the human visual perception with the variable values, different fuzzy sets were defined: “Very Low” (VL), “Low” (L), “Normal” (N), “High” (H) and “Very High” (VH).

**Figure 9:**
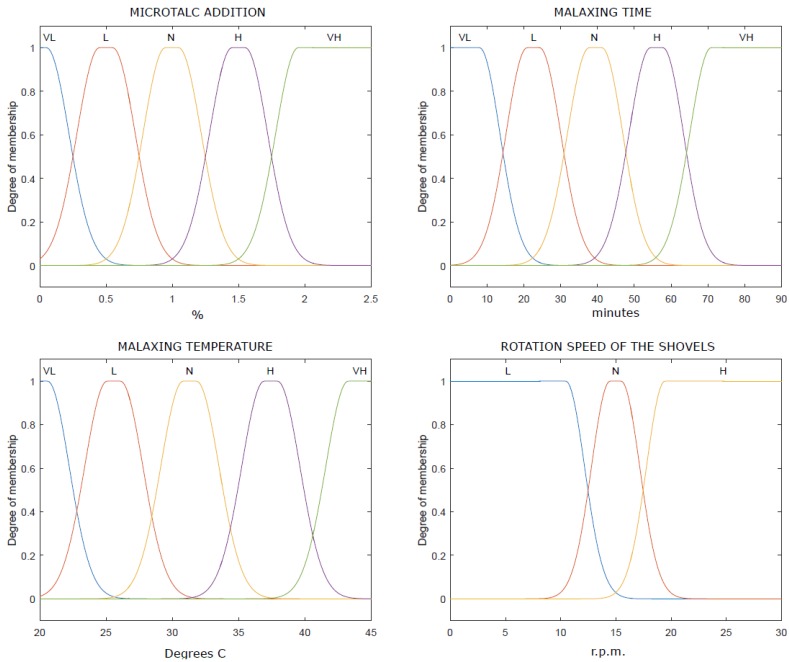
Output fuzzy membership functions.

The membership functions of the output variables are shown in Figure 9. They were configured as follows:

- Mt: The malaxing time variable denotes the amount of time the olive paste is stirring inside the thermomixer. The universe of discourse of this variable is between the minimum value of time needed to prepare the olive paste (around 20 min) and the maximum value of time to avoid formations of emulsions (close to 90 min) [12]. The malaxing time in the thermomixer can be modified by acting on the olive paste outflow.
- MT: The malaxing temperature of reference ranges between 20 °C and 40 °C [12]. Usually the temperature of the paste can be controlled by acting in the servo-valve that regulates the inflow of hot water.
- C: Coadjuvant addition is done by pumping from the container. In this study, has been considered that microtalc percentage varies from 0% to 2% [12], depending on the type of pasta.
- S: Shovel speed. Normally, its value ranges between 10 and 20 rpm. This value needs to be adjusted to the minimum with difficult pastes for not favoring the appearance of emulsions [12].

Finally, the combinational logic rules were defined according to the common operation mode where the elaboration objective is to maximize the quantity of olive oil produced without loss of quality. They were formulated as shown in Table 1.

## 3. Experimental Results

**Figure 10:**
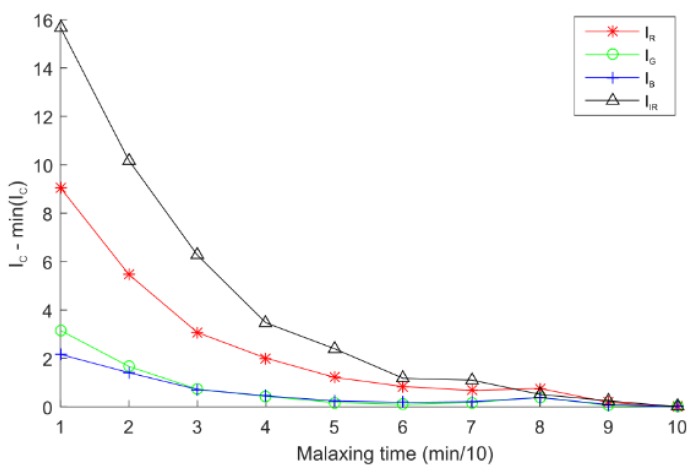
Viscosity index for different channels versus malaxing time.

The viscosity index was evaluated from the color intensity index $\bar{I_{c}}$ computed for each image channel by monitoring the quantity of olive paste attached to the shovels. Figure 10 represents the $\bar{I_{R}}$, $\bar{I_{G}}$, $\bar{I_{B}}$ and $\bar{I_{IR}}$ parameters versus the malaxing time for the red, green, blue, and infrared channels respectively. As the quantity of paste attached to the shovel decreases, the $\bar{I_{c}}\;$index decreases. The results prove that the infrared channel was more sensitive than the rest of the channels. The slope was negative to the point number six when there are no visual changes.

**Figure 11:**
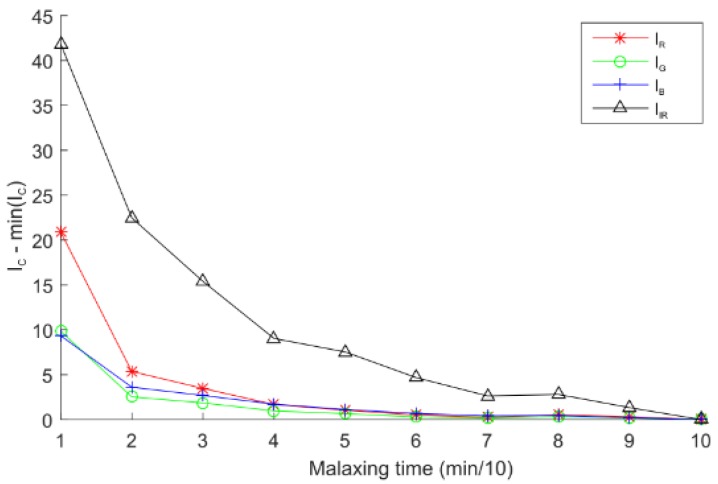
Floating oil index for different channels versus malaxing time.

Furthermore, the floating oil index was achieved by monitoring the olive paste surface inside the thermomixer. Also, the average intensity for each channel was computed. As in the viscosity index case, the results show that the infrared channel ($\bar{I_{IR}}$) was more sensitive than other channels to the presence of floating oil over the paste. The differences between channels can be clearly seen in Figure 11.

The textural parameters $C_{c,n,m}$, $E_{c,n,m}$ and $H_{c,n,m}$ were individually computed for each image. Then the *m* dimension was removed by averaging the values which are related to the same sample. After that, these parameters were scaled from 0 to 1 to compare the resultant values. Finally, the lineal relation between the textural parameters and the malaxing time was evaluated by fitting the different sequences (*n* from 1 to 12) with a one-degree polynomial. This procedure was repeated for each color channel *c*.

**Figure 12:**
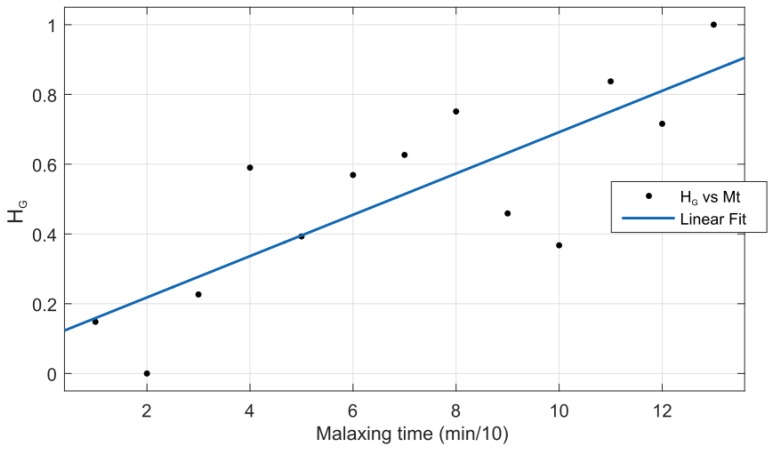
Homogeneity parameter in the green channel versus malaxing time.

Table 2 presents the results. It shows that the best correlation was reached with the homogeneity parameter in the green channel ($H_{G}$) with the least value of root mean square error. Figure 12 presents this linear relationship. It means that the GLCM matrix is skewed towards its diagonal of the matrix according to the malaxing time increment. This could be due to the lumps over the olive paste surface tending to be larger at the end of the malaxing time when olive paste can be considered as prepared. Similar results were obtained in [13] where authors employed computer vison and the GLCM matrix to assess when the dough for the bakery was ready. They reached linear dependency between the homogeneity parameter and the specialist knowledge.

All the aforementioned indexes were coded according to the fuzzy levels (Figure 8) and membership thresholds were validated with the master miller knowledge through interviews. A total of 100 images with high, intermediate, and low indexes were presented to the master miller and his assessment was compared with our approach. The results yielded an average of 90% of success ratio.

**Figure 13:**
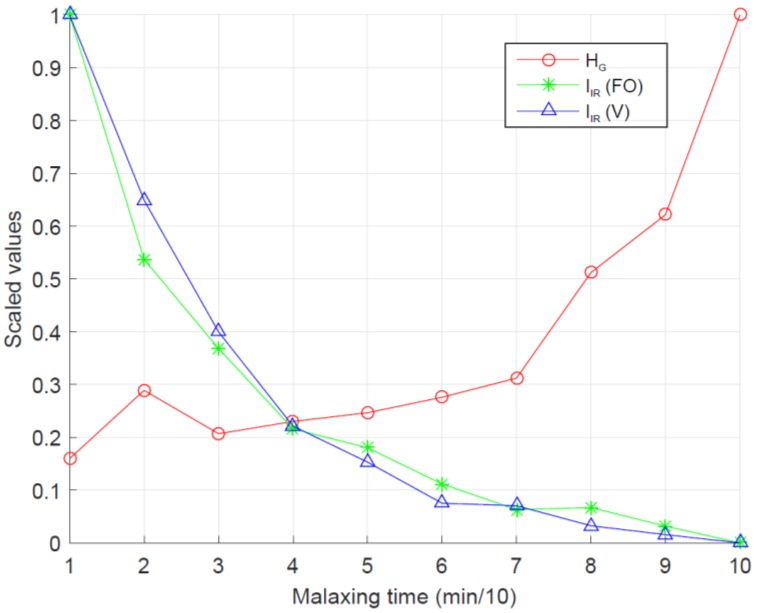
Inputs values for the controller where $H_{G}$ is the textural parameter homogeneity in the green channel, $I_{IR}$ (FO) is the intensity value of the infrared channel for floating oil and $I_{IR}$ (V) is the same value for viscosity

**Figure 14:**
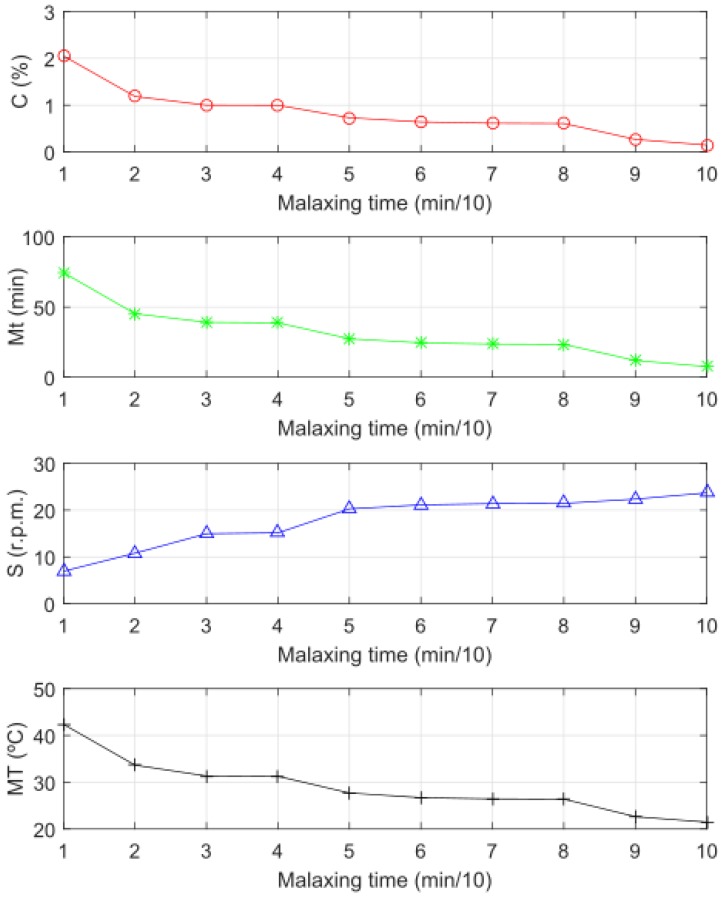
Outputs for different olive paste conditions.

Finally, the $H_{G}$, $I_{IR}$ (floating oil) and $I_{IR}$ (viscosity) parameters were considered as controller inputs (Figure 13) and the outputs were evaluated according to the sequence of images acquired during the malaxing process. For each malaxing state the system recommended four set points (*C*, *Mt*, *S* and *MT*) (Figure 14). For example, to prepare difficult pastes, the system recommended high temperature, high addition of microtalc, long malaxing time and low rotating speed of the shovels. The variables of temperature, microtalc addition and malaxing time were decreased and the speed of the shovels was increased as the paste tends to be prepared.

## 4. Conclusions

In this work a new control system for the olive oil malaxing process has been designed to evaluate the olive paste and to act accordingly. To determinate the olive paste conditions a malaxing state sensor has been developed. It was composed by a vision camera device and different processing algorithms to extract features from the acquired images. These features were floating oil, viscosity, and granularity. The infrared channel ($I_{IR}$) revealed more sensitivity for the determination of floating oil and viscosity. On the other hand, the textural parameter homogeneity in the green channel ($H_{G}$) showed the best correlation with the evolution of the olive paste granularity. From these parameters and the control miller expertise a fuzzy logic controller was implemented. Different technological variables were considered as controller outputs, such as malaxing time, malaxing temperature, addition of coadjuvant and rotating speed of the shovels. Furthermore, the controller was evaluated from a sequence of images acquired from a real plant and it obtained the expected reference values of the involved variables in the thermomixer. The configured system allows detection of difficult pastes, and allows the adaptation of the malaxing process to its features. Thus, it avoids olive oil leak in the pomace due to non-prepared olive paste and it suggests a significant improvement in the energy consumption of the plant.

## Figures and Tables

**Table 1:** Combinational logic rules.

IF	THEN
V is VL & FO is VH & G is VH	S is H & MT is VL & Mt is VL & C is VL
V is L & FO is H & G is H	S is H & MT is L & Mt is L & C is L
V is N & FO is N & G is N	S is N & MT is N & Mt is N & C is N
V is H & FO is L & G is L	S is L & MT is H & Mt is H & C is H
V is VH & FO is VL & G is VL	S is L & MT is VH & Mt is VH & C is VH

**Table 2:** Textural parameters.

Parameter	Mean	SD	R^2^	RMSE
C_R_	0.44	0.29	0.48	0.21
C_G_	0.46	0.27	0.54	0.18
C_B_	0.51	0.28	0.35	0.22
C_IR_	0.61	0.26	0.49	0.19
E_R_	0.41	0.27	0.37	0.21
E_G_	0.28	0.30	0.51	0.20
E_B_	0.32	0.28	0.13	0.26
E_IR_	0.41	0.27	0.42	0.20
H_R_	0.50	0.30	0.56	0.19
H_G_	0.51	0.28	0.62	0.17
H_B_	0.46	0.27	0.38	0.21
H_IR_	0.38	0.26	0.54	0.17
